# An Account of a Curious Fact Relative to the Effects of Crude Mercury

**Published:** 1787

**Authors:** Michael Underwood

**Affiliations:** Physician to the British Lying-in Hospital, and Licentiate in Midwifery of the Royal College of Physicians, London


XI. *An Account of a curious Fact relative to the Effects of crude Mercury. Communicated in a Letter to Dr.* Simmons *by* Michael Underwood, *M. D. Phyfician to the Britifh Lying-in Hofpital, and Licentiate in Midwifery of the Royal College of Phyficians, London.*

ALTHOUGH the internal exhibition of crude mercury has not been without its advocates, ever fince its introduction into medicinal dicinal ufe, yet it was confined to a very fmall number of difeafes, till the time of Dr. Dovar. His account of its virtues ought, perhaps, to be taken with confiderable limitations; but the moft eminent phyficians fince his time have been in the habit of prefcribing it, in common with other uncertain remedies, in many complaints difficult of cure, or ill underftood. In fuch cafes, it has in many inftances proved eminently ferviceable; but though generally accounted perfectly innoxious, fome phyficians have entertained fcruples in this refpect, and have argued, a priori, that it muft be likely, from different caufes, to be abforbed in its paffage through the primæ viæ, and to raife a falivation; as the æthiops mineralis, and even the mercurius alcalizatus have, in fome inftances, been reported to do. I know of no fuch inftance, however, upon record, with regard to the crude mercury, and on that account prefume the following fact may appear to you of fufficient importance to merit a place in your Medical Journal.

A clergyman of my acquaintance, who for more than thirty years has been afflicted with afthma, and at different times been thought unlikely unlikely to furvive many days, above twenty years ago having been advifed to take crude mercury, received great benefit from it, and, I believe, is indebted to it for the prefervation of his life; for I have known him recovered from feveral very violent attacks by a fteady adherence to the ufe of this remedy. After repeated inftances of this kind, he was fo much in the habit of taking it, that above two years ago he had fwallowed to the amount of upwards of an hundred weight of quickfilver.

Since that time, the improved ftate of his health, though he is now upwards of fixty years of age, having enabled him to decline the ufe of his remedy for feveral months, he happened to be feized with an intermittent, and had recourfe to the powder of bark, which he took in large dofes. While he was under this courfe, a friend, who had been lately feized with afthma, happening to call upon him, the clergyman advifed him to make a trial of his favourite medicine; and his friend upon hearing fuch a fatisfactory account of it was ready enough to comply: but inquiring, with folicitude, how he might be able to fwallow fo unmanageable a fluid, the clergyman very readily furnifhed him with the beſt directions, by ſwallowing an ounce of it in his preſence. The conſequence of this friendly recommendation of his catholicon, was a ſalivation, which took place in about eight and forty hours, and continued very ſeverely for eight or ten days, producing foul and painful ſores in the mouth, and eſſentially impairing his health.

How far the large quantity of the bark he had taken, or any other circumſtances, might contribute to occaſion ſuch a change in the effect of a medicine that uſually makes its way without any ſenſible operation, I ſhall not venture to inquire; but content myſelf with communicating to you a curious fact, without attempting to explain it.

*Great Marlborough Street,*

Feb. 26, 1787.

XII. *An*

